# Uncovering the impact of UV radiation on mitochondria in dermal cells: a STED nanoscopy study

**DOI:** 10.1038/s41598-024-55778-z

**Published:** 2024-04-15

**Authors:** Hyung Jun Kim, Seon-Pil Jin, Jooyoun Kang, So Hyeon Bae, Jung Bae Son, Jang-Hee Oh, Hyewon Youn, Seong Keun Kim, Keon Wook Kang, Jin Ho Chung

**Affiliations:** 1 Department of Chemistry, Seoul National University, Seoul, 08826 South Korea; 2 Institute of Radiation Medicine, Medical Research Center, Seoul National University, Seoul, 03080 South Korea; 3 Department of Dermatology, Seoul National University Hospital, Seoul, 03080 South Korea; 4 Department of Dermatology, Seoul National University College of Medicine, Seoul, 03080 South Korea; 5 Institute of Human-Environment Interface Biology, Medical Research Center, Seoul National University, Seoul, 03080 South Korea; 6 Department of Nuclear Medicine, Seoul National University Hospital, Seoul, 03080 South Korea; 7 Department of Nuclear Medicine, Seoul National University College of Medicine, Seoul, 03080 South Korea; 8 Cancer Research Institute, Seoul National University College of Medicine, Seoul, 03080 South Korea; 9 Department of Biophysics and Chemical Biology, Seoul National University, Seoul, 08826 South Korea; 10 Department of Biomedical Sciences, Seoul National University College of Medicine, Seoul, 03080 South Korea; 11 Bio-MAX Institute, Seoul National University, Seoul, 08826 South Korea

**Keywords:** Nanobiotechnology, Cellular imaging

## Abstract

Mitochondria are essential organelles in cellular energy metabolism and other cellular functions. Mitochondrial dysfunction is closely linked to cellular damage and can potentially contribute to the aging process. The purpose of this study was to investigate the subcellular structure of mitochondria and their activities in various cellular environments using super-resolution stimulated emission depletion (STED) nanoscopy. We examined the morphological dispersion of mitochondria below the diffraction limit in sub-cultured human primary skin fibroblasts and mouse skin tissues. Confocal microscopy provides only the overall morphology of the mitochondrial membrane and an indiscerptible location of nucleoids within the diffraction limit. Conversely, super-resolution STED nanoscopy allowed us to resolve the nanoscale distribution of translocase clusters on the mitochondrial outer membrane and accurately quantify the number of nucleoids per cell in each sample. Comparable results were obtained by analyzing the translocase distribution in the mouse tissues. Furthermore, we precisely and quantitatively analyzed biomolecular distribution in nucleoids, such as the mitochondrial transcription factor A (TFAM), using STED nanoscopy. Our findings highlight the efficacy of super-resolution fluorescence imaging in quantifying aging-related changes on the mitochondrial sub-structure in cells and tissues.

## Introduction

In recent decades, far-field optical microscopy has emerged as a primary technology for structurally and functionally studying cellular components^1^. Super-resolution optical microscopy techniques have facilitated the study of biological systems at the molecular level by overcoming the optical resolution limit of ~ 200 nm imposed by diffraction while also avoiding the challenges posed by other high-resolution techniques such as intricate sample preparation and limited imaging area^2,3^. Molecular components in cells are typically smaller than the resolution limit of conventional microscopy, which remarkably limits the ability to study subcellular protein distributions. Therefore, super-resolution imaging is the most suitable method for qualitatively and quantitatively analyzing the distribution of proteins in many cellular organelles. Furthermore, super-resolution microscopy has recently shown potential for exploring the undeveloped nanoscale regime in tissues kept in long-term storage for biological specimens^4^.

Mitochondria, as the powerhouse of the cell, are vital organelles in indispensable lipid membrane metabolism pathways such as ATP production^5^. In light of deleterious biological influences, the purview of mitochondrial function has been broadened to encompass not only its involvement in the respiratory chain but also its implication in the pathogenesis of age-related maladies and neurodegenerative disorders, including but not limited to Alzheimer’s and Parkinson’s disease^6,7^. The defects in the oxidative phosphorylation system assembly on the cristae of mitochondria give rise to numerous serious human diseases^8^. Being the primary manufacturers of both cellular energy and free radicals, dysfunctional mitochondria are particularly thought to induce a considerable decline in critical muscle function^9^. Moreover, as the biological effect of mitochondria is linked to aging, mitochondrial dysfunction in the electron transport chain involving free radical generation has been implicated in the aging process. Recent observations have suggested that the aging-dependent decrease of mitochondrial complex II, a mitochondrial complex associated with the electron transport chain, activity in human dermal fibroblasts is involved in the aging process^10^. Consequently, progressive accumulation of oxidative damage, mostly occurring due to mitochondrial dysfunction, is one of the considerable factors for aging^11^.

UV irradiation can trigger skin aging in the long term, particularly for the skin, which displays visible signs of aging as people grow older. Skin aging, characterized by wrinkle formation, loss of elasticity, and dryness, is divided into two major processes: (1) natural aging, also known as chronological aging, and (2) photoaging^12^. Photoaging is a process that accelerates natural aging through repeated ultraviolet (UV) exposure^13,14^. Mitochondrial dysfunction is one of the detrimental effects of UV irradiation that contributes to photoaging^15^. For instance, UV irradiation can cause structural changes in mitochondria such as mitochondrial outer membrane fragmentation and mitochondrial DNA damages, resulting from oxidative stress^15,16^. Therefore, we propose to use dermal cells and tissues exposed to acute or chronic UV radiation as a model system to monitor the accumulation of mitochondrial changes associated with accelerated aging and/or the aging process^17^.

As a means to monitor aging-related responses, mitochondria and their relevant molecular components are ideal targets^18^. Mitochondria form a dynamic reticulum of long, branched tubular networks that contain extensive biomolecular distributions and spread them throughout the cytoplasm of the cells, depending on the cell type and environmental conditions^19^. In eukaryotes, oxidative phosphorylation occurs in mitochondria, which are organelles bound by inner and outer membranes that surround a dense matrix containing enzymes involved in intermediate metabolism and numerous copies of a genome that encodes partial proteins on the inner membrane and the RNAs required for their translation^20^. To maintain the quantitative and structural balance in mitochondria, fission and fusion play crucial roles in maintaining the biological functions of mitochondrial molecules during metabolic reactions or environmental stresses. Fission is necessary for producing new mitochondria and can also help to remove damaged mitochondria and induce apoptosis under extreme cellular stress. Fusion reduces stress by mixing the components of partially damaged mitochondria as part of the complementation process^21^. Therefore, establishing methods to quantify mitochondrial morphology can provide a means to develop evaluation methods for mitochondrial alteration by oxidative stress^16^.

Efforts to understand the molecular components that determine mitochondrial structure and their connections with extra-mitochondrial pathways in various cell types have led to the application of super-resolution techniques in mitochondrial research^6,22^. Using 3D STORM, researchers were able to visualize the structure of the mitochondrial network and the spatial relevance between mitochondria and microtubules in cells^23^. In another study, a group of researchers found that a core subunit of the mitochondrial inner membrane organizing system (MINOS in short) is localized at the junctions of cristae and that its clusters show an ordered distribution on the mitochondrial inner membrane that depends on the location of the mitochondria in the cell and on the orientation of the growing cellular surface using stimulated emission depletion (STED) nanoscopy^24^. By mapping Percoll-purified mitochondrial proteins from murine hearts using STED, they were able to quantify and resolve distinct protein clusters within mitochondria^25^.

Mitochondria play a central role in maintaining cellular energy metabolism and various cellular functions, and their dysfunction is related to UV-induced cellular damage and photoaging^26–28^. While the overall architecture and major functions of mitochondria have been described progressively, the relationship between mitochondria and aging remains poorly understood. In this study, we investigated the feasibility of using super-resolution STED nanoscopy as a tool for estimating UV-mediated variation through visualizing and quantifying the morphological changes of mitochondrial molecules in sub-cultured human primary skin fibroblasts and mouse skin tissues. Our results demonstrate the potential of super-resolution imaging to shed light on the relationship between mitochondria and photoaging by resolving the UV-induced morphology changes of these molecules at the sub-diffraction limit.

## Results and discussion

### Optimizing UV irradiation conditions to induce mitochondrial changes in skin primary fibroblasts without cell apoptosis

Chronic UV exposure leads to skin folds and extracellular matrix damage, which precedes photoaging, characterized by the induction of matrix metalloproteinase (MMP-1) and the reduction of procollagen synthesis^29^. Since accumulation of UV damage, rather than acute cell death, results in photoaging, we found a subtoxic dose of UV irradiation to the cells while having other substantial effects. Specifically, in our *in-vitro* experiment, we observed that UV irradiation decreased the protein level of procollagen and increased MMP-1 proteins in normal human dermal fibroblasts (NHDFs), depending on the irradiation dose (Supplementary Figure S1a). Moreover, increasing UV doses altered the structure of PARP-1 enzymes involved in DNA repair, as the cleavage form, which is regarded as a hallmark of apoptosis, by activated caspases^30^ (Supplementary Figure S1b). Therefore, we determined that 100 mJ/cm^2^ of UV irradiation was sufficient to produce aging-related results while minimizing cell apoptosis (Supplementary Figure S1).

Previous research has shown that apoptotic stimuli, such as UV irradiation, can trigger changes in the mitochondrial matrix and result in the release of cytochrome c^31^. Additionally, the activity of mitochondria could be adapted to changing cellular conditions. It has been suggested that short-term variations in energy demand may be compensated without modifying the mitochondrial enzyme contents, but modulation of the mitochondrial protein content has been observed during long-term adaptations^2,32^. Thus, we aimed to simultaneously observe the overall change in mitochondrial structure and enzymatic changes related to apoptosis under UV irradiation. Our results showed that the UV stimulus increased the fragmented ratio of mitochondria from filamentous to spherical shape and the cleaved form PARP-1. Notably, at 30 min after 100 mJ/cm^2^ UV treatment, we observed a decrease in mitochondrial length, as defined by outer membrane morphology, without cell death. However, exposure to UV over 200 mJ/cm^2^ resulted in both morphological changes in mitochondria and cell apoptosis (Supplementary Figures S1b and S2).

### Qualitative and quantitative characterization of TOM20 distribution on the mitochondrial outer membrane using STED nanoscopy

**Figure 1:**
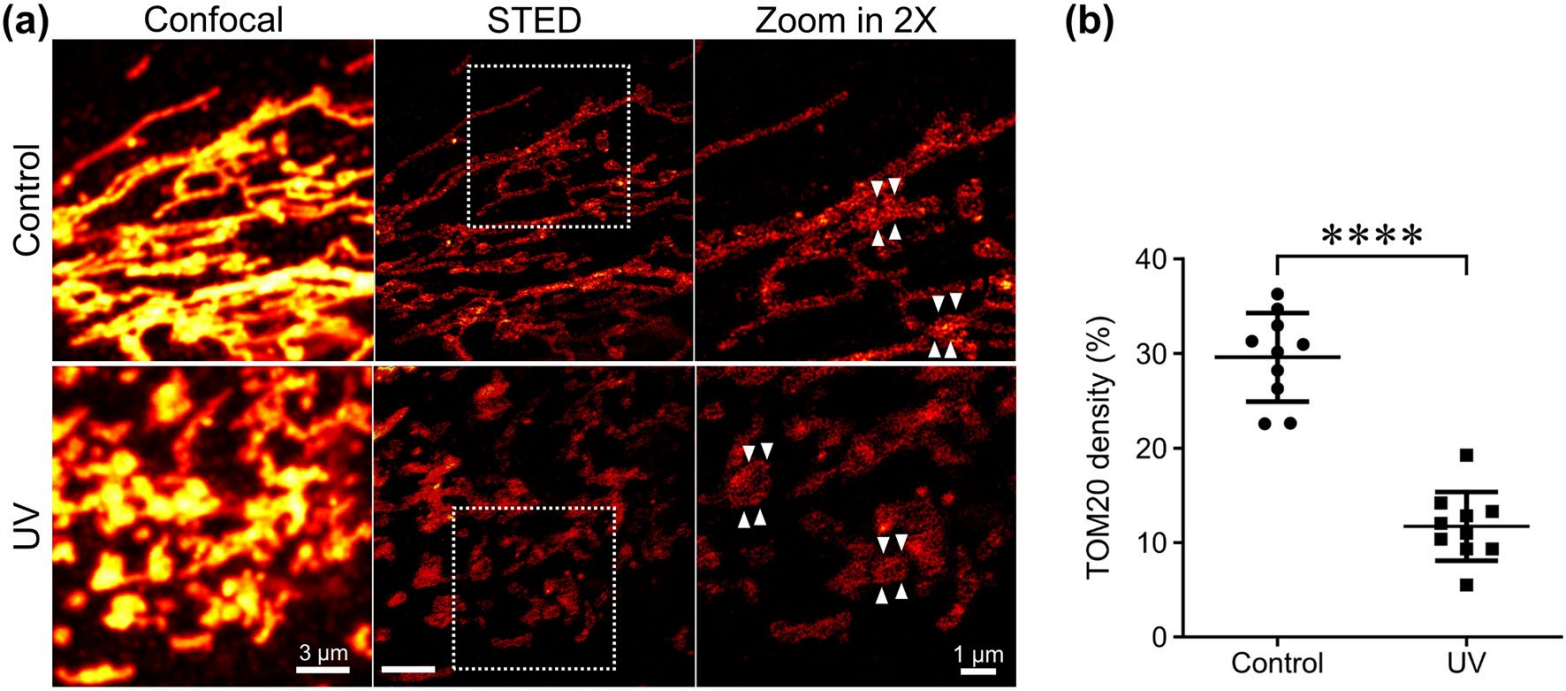
UV irradiation induces the distribution change of mitochondrial membrane proteins. (**a**) At each condition, the cells were fixed and labeled with anti-TOM20 antibody. Images were obtained by confocal and STED microscopy. (**b**) The density of TOM20 clusters was quantitatively analyzed in each region of interest (ROI). Ten ROIs were included in each group for generating quantitative analysis plots. The data are depicted as mean ± SD. ****p < 0.0001.

The translocase of the outer membrane (TOM) complex serves as the central entry gate for most nuclear-encoded mitochondrial proteins^2,33–35^, with TOM20 being the initial recognition site for preproteins on the outer mitochondrial membrane. This facilitates protein movement and transfers preproteins to the central receptor, TOM22^36–39^. Thus, monitoring changes in the distribution of TOM20 is essential to capture the alteration of the mitochondrial outer membrane. Although confocal microscopy with anti-TOM20 staining provides an overall view of mitochondrial morphology, its resolution is limited due to diffraction limits and the narrow distance between targets (left panel in Fig. 1a). Moreover, regardless of staining methods, both the mitochondria labeled with anti-TOM20 and the mitochondria stained with MitoTracker appear as interconnected tubular structures, spanning a size range from a few microns to sub-20 microns^19^. In contrast to the images presented by confocal fluorescence microscopy, STED nanoscopy enables the visualization of individual TOM20 complexes as isotropic-shaped clusters (the middle and right panels in Fig. 1a).

We conducted a detailed comparison of representative STED images of TOM20 clusters between two groups of fibroblast cells. The average diameter of TOM20 clusters was ~ 100 nm (Supplementary Figure S3). Because the clusters were decorated using the primary and secondary antibodies and the unit pixel size was 50 nm, the clusters appeared larger than their actual size. The broadness of the size distribution for TOM20 clusters was similar regardless of UV exposure. However, the density distribution of TOM20 clusters on mitochondria significantly decreased upon UV irradiation (Fig. 1b). Moreover, the clusters in UV-treated fibroblasts were localized at the rim of the mitochondrial outer membrane. Our results suggest that super-resolution STED nanoscopy can be used to observe the nanoscale distribution of TOM20 clusters and evaluate the quantitative changes in cluster density induced by acute aging-related stimulation. The reliability of our finding is supported by a previous study from the Hell group, which suggests that the nanoscale distribution of translocase clusters may vary depending on the functional requirements of mitochondria in different cell lines^2^.

To validate our analytical methodology based on super-resolution fluorescence imaging, we conducted transmission electron microscopy (TEM) measurements via immunogold staining to compare the TOM20 distribution on cryo-ultramicrotome cut sections of cells prepared using the same method as that used for fluorescence imaging (Supplementary Figure S4). The quantitative changes observed on TEM images were consistent with those on our STED images. However, compared to STED nanoscopy, TEM is a more complex technique, requiring intricate sample preparation and may cause damage to the specimen (e.g., the mitochondrial membrane and immunostained gold beads) due to the high voltage electron. Additionally, TEM images only provide information on TOM20 clusters on a narrow cutting plane and do not give a three-dimensional view of the mitochondrial outer membrane.

The decrease in TOM20 complex distribution observed through STED nanoscopy implies that UV exposure may inhibit biomolecular migration between the mitochondria and their surroundings, such as the cytosol, leading to latent changes in mitochondrial membrane potentials. To support this hypothesis, we compared the relative fluorescence intensity of mitochondria stained with MitoTracker Deep Red FM, a membrane potential sensitive dye, using confocal microscopy. Supplementary Figure S5 shows that the fluorescence intensity was significantly reduced after UV irradiation, indicating a change in mitochondrial membrane potential.

### Quantifying mitochondrial transcription factor A, as an aging-related factor, on the single cell level using STED nanoscopy

The mitochondrial transcription factor A (TFAM) protein is a key component of nucleoids in mitochondria, and its functions include binding, wrapping, bending, and unwinding mtDNA in mammalian cells, analogous to the movement of other high-mobility group box (HMGB) proteins^40,41^, as well as playing a critical role in the initiation of mitochondrial transcription and replication^42^. A loss of mitochondrial DNA and embryonic lethality are observed in cases where the TFAM gene has been knocked out^43,44^. Moreover, its expression is known to change significantly with aging, making it a pertinent target for aging research^45^.

**Figure 2:**
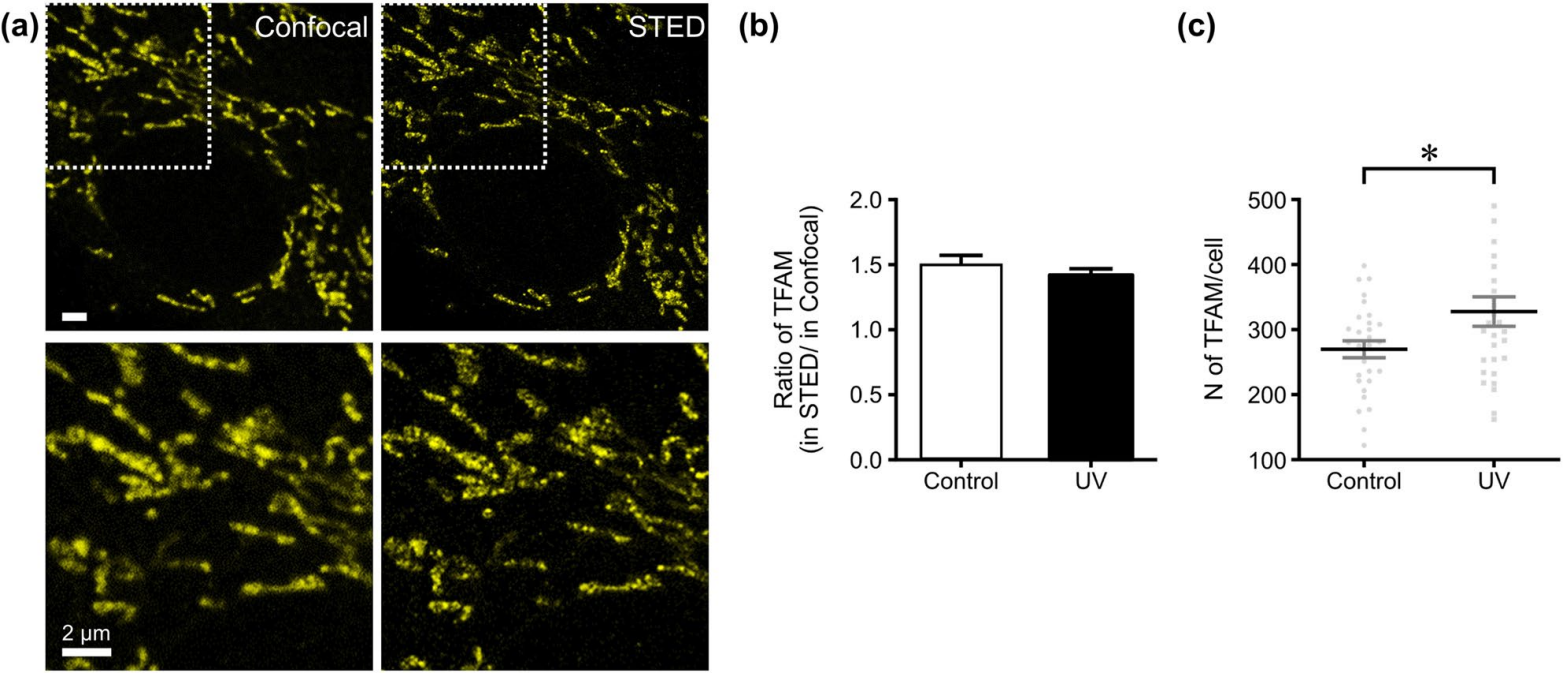
STED nanoscopy precisely resolves the distribution of TFAM molecules. (**a**) At each condition, the cells were fixed and labeled with anti-TFAM antibody. Images were obtained by confocal and STED microscopy. (**b**) STED provides a ~ 1.5 times more accurate number of TFAM molecules as compared to conventional confocal microscopy in the cells. (**c**) The distribution of TFAM was quantitatively analyzed in each cell. More than 30 cells were included in each group for generating quantitative analysis plots. The data are depicted as mean ± SD including the value for each cell. *P < 0.03.

Initially, confocal microscopy was used to image mitochondrial nucleoids labeled with TFAM antibodies in skin primary fibroblasts. However, this approach presented only a sparse pattern of nucleoids on the mitochondrial membrane network and indistinguishable TFAM locations within the diffraction limit (left panel in Fig. 2a). In confocal images, nucleoids that are located in close proximity and within the diffraction limit are observed as a single blurred spot, despite the fact that their true size is less than approximately 250 nm. However, STED nanoscopy enabled us to examine the size of the nucleoids and their distribution. The application of STED nanoscopy in this study revealed a diminutive size of nucleoids relative to that observed with confocal microscopy. Individual nucleoids could be distinguished using STED nanoscopy (right panel in Fig. 2a), with an average diameter of ~ 100 nm, which is congruent with the actual size of antibody-labeled nucleoids. It is also similar to the average size of mitochondrial nucleoids in mouse fibroblasts (~ 110 nm), as previously determined by studies using PALM or dSTORM with fluorescent-tagged TFAM or mtDNA antibodies^43,46^. Thus, our results demonstrate that super-resolution microscopy is capable of identifying the uniform size of mitochondrial nucleoids in mammalian cells^40^.

Given that TFAM is a marker of mitochondrial nucleoids and closely linked to aging, we quantitatively analyzed the number of TFAMs per cell in fibroblast cells. Our results showed that the average number of nucleoids observed by STED nanoscopy was ~ 1.5 times higher than that observed by confocal microscopy (Fig. 2b). Furthermore, the average number of TFAM increased after UV irradiation, consistent with the results obtained through western blot analysis as a typical quantitative analysis (Fig. 2c and Supplementary Figure S6).

### Quantitative analysis of mitochondrial translocase proteins in paraffin-embedded tissues with STED nanoscopy

**Figure 3:**
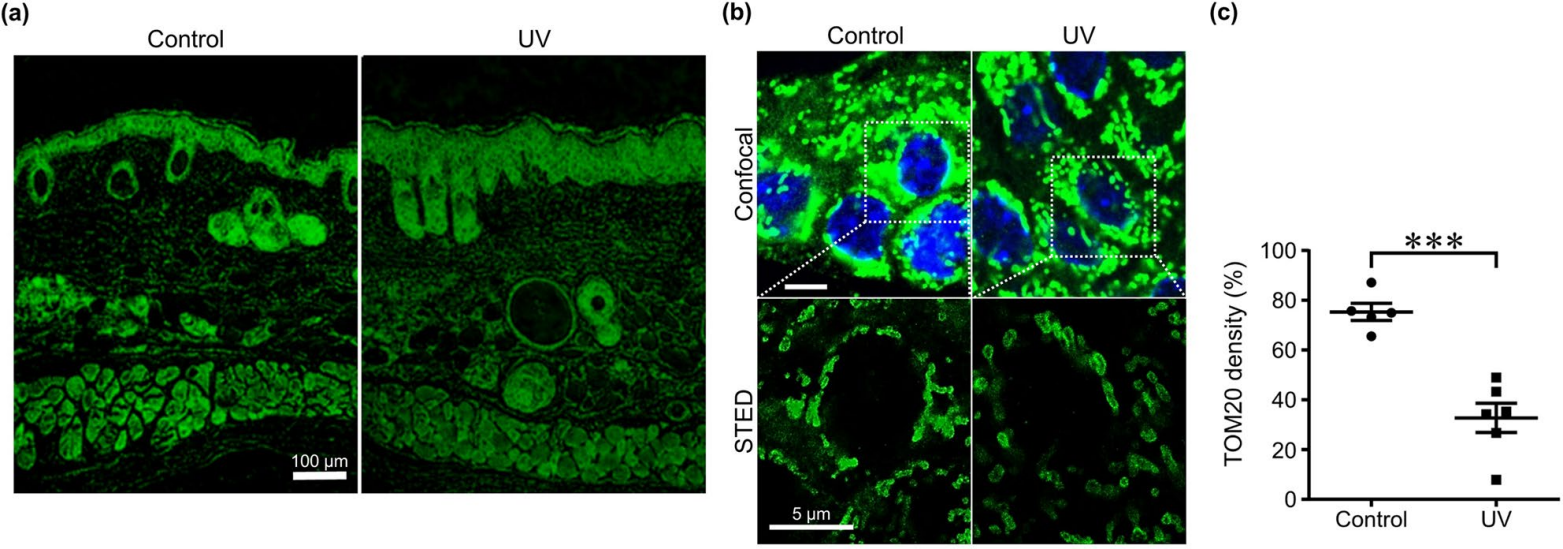
Quantitative analysis of TOM20 clusters on mice skin tissues using STED nanoscopy. (**a**) UV irradiation was applied three times a week for 8 weeks to induce chronic UV damaged skin (photoaged mice skin model). Dorsal back skin was harvested 72 h after the last UV irradiation. Skin tissues were fixed and then paraffin embedded for further analysis. Immunofluorescence staining was performed using anti-TOM20 antibody. (**b**) Epidermal layer, the outermost compartment of the skin, was closely examined through both confocal and STED microscopy (**c**) TOM20 cluster densities can be quantitatively analyzed from STED images. (*n* = 5–6 mice) *** *p* < 0.0002.

To evaluate the suitability of paraffin-embedded tissues for fluorescence imaging, we attempted to label both targets on the *in-vivo* photoaged samples and imaged them using confocal microscopy. While staining with the TOM20 antibody was successful in all mouse skin tissues, the TFAM antibody did not yield any results. Despite this setback, we were able to observe mitochondrial proteins in three representative layers of the tissues, consisting of keratinocytes in the epidermis, fibroblasts in the dermis, and myocytes in the panniculus carnosus (Fig. 3a). However, in contrast to the *in-vitro* experiments with fibroblasts, confocal microscopy failed to discern any morphological changes in the outer membrane of mitochondria in tissues. For instance, while we observed that UV irradiation shortened the broad spread of mitochondrial outer membrane morphology from nuclei in cultivated skin primary fibroblasts, the morphology in the tissue showed a similar pattern regardless of chronic exposure to UV irradiation due to the three-dimensionally dense packing structure in tissues. Nonetheless, even though only one antibody works, multicolor fluorescence imaging was possible on paraffin-embedded tissues, as the blue signal from DAPI highlighting the nuclei was detected in all tissue area (top panel in Fig. 3b).

To perform nanoscale analysis in tissues, we applied STED nanoscopy (Fig. 3b). The magnified STED images showed that TOM20 had partially spherical cluster shapes within the mitochondria, but the mitochondrial distribution pattern was still comparable to that observed in cultivated skin primary fibroblasts in terms of the density of the TOM20 distributions. We quantitatively compared the distribution density of TOM20 clusters and found that TOM20 clusters were evenly spread on mitochondria of keratinocytes in normal mouse skin tissues, whereas there was a reduced amount of TOM20 clusters in tissues chronically exposed to UV radiation (Fig. 3c). This trend also appeared in the fibroblasts in the dermis of tissues. These results suggest that paraffin-embedded tissues have structural preservation on the nanoscale, making STED super-resolution nanoscopy a suitable method for analyzing the nanoscale distribution of aging-related proteins in tissues stored by the standard storage method.

Photoaging, which is a hallmark of chronically sun-exposed skin, has been recently investigated using advanced macroscopic and microscopic technologies to unravel its molecular mechanisms. We aimed to assess the utility of STED nanoscopy as a tool for analyzing the degree of aging in dermal cells. Using super-resolution STED nanoscopy, we were able to identify individual mitochondrial protein molecules and perform a more precise quantitative analysis of TOM20 and TFAM molecules, compared to conventional confocal microscopy. Additionally, we demonstrated that our highly resolved fluorescence imaging could differentiate the distribution of mitochondrial translocases between normal tissues and those chronically exposed to UV radiation.

Further improvements are needed to establish this method as a universal technology for assessing biological aging, such as developing automated super-resolution imaging systems and image analysis algorithms^47^. Nonetheless, this study paves the way for the potential use of super-resolution imaging as a tool for quantitative analysis of aging-related proteins in living cells. This could have significant implications for the development of new strategies for photoprotection and repair of photodamaged human skin^16,48–50^.

## Materials and methods

All study materials were approved by an appropriate institution (Institutional Review Board or Institutional Animal Care and Use Committee). In addition, all experiments were performed in accordance with relevant guidelines and regulations.

### Cell culture and UV irradiation

Human tissues for primary cell culture were obtained under written informed consent and in accordance with an approved protocol by the Institutional Review Board of Seoul National University Hospital. NHDFs were isolated from foreskin tissue and cultured in Dulbecco’s Modified Eagle’s Medium (DMEM) (Welgene, Gyeongsan, South Korea) supplemented with 10% fetal bovine serum (FBS) (Hyclone, Logan, UT, USA) and 1% streptomycin/penicillin (Life Technologies, Rockville, MD, USA). The cells were incubated in a humidified incubator with 5% CO_2_ at 37 °C. For all experiments, cells were used between the fifth and tenth passages of fibroblasts. Prior to UV irradiation, fibroblasts were serum-starved overnight in DMEM with 0.2% FBS. Cells were washed with phosphate-buffered saline (PBS) and irradiated with an indicated dose (mJ/cm^2^) of UV in PBS. TL 20W/12 RS fluorescent lamps (Philips, Eindhoven, Netherlands) with an emission spectrum between 275 and 380 nm (*λ*_max_: 310–315 nm) were used for UV irradiation. The power output distribution of the UV emission spectrum was 11.2% UVA2 (340–380 nm), 25.3% UVA1 (320–340 nm), 53.3% UVB (290–320 nm), and 10.2% UVC (275–290 nm). A Kodacel filter (TA401/407) (Kodak, Rochester, NY, USA) was used to block wavelengths below 290 nm (UVC range). The UV irradiance was measured with a UV meter (Model 585 100) (Waldmann, Villingen-Schwenningen, Germany)^51,52^. After UV irradiation, PBS was replaced with DMEM with 0.2% FBS, and the cells were further incubated for the specified time periods.

### UV irradiated cell staining

The fibroblasts were fixed with paraformaldehyde and stained. For mitochondrial imaging, we chose translocases of the TOM20 and TFAM as the target molecules. Polyclonal rabbit antibodies against TOM20 from Santa Crus Biotechnology and polyclonal mouse antibody against TFAM from Abnova were used as primary antibodies for labeling mitochondrial proteins. Subsequently, Alexa488 conjugated secondary antibodies (Anti-mouse-IgG-Alexa488 from Jackson ImmunoResearch Laboratories) and STAR635P conjugated secondary antibodies (Anti-rabbit-IgG-Star635P from Abberior) were used for fluorescence detection. After immunolabeling, the samples were mounted in Prolong Gold with DAPI for nucleus staining (Invitrogen, Grand Island, NY, USA).

### Tissue preparation and staining

Animal studies were performed in accordance with the ARRIVE guidelines. To establish a photoaging model, SKH-1 hairless mice aged six weeks were exposed to chronic UV irradiation using the same UV lamps employed in the cellular experiments. Mice received UV exposure three times per week on Mondays, Wednesdays, and Fridays for a total of eight weeks. The irradiation dose was gradually increased by one minimal edema dose (1 MED = 100 mJ/cm^2^) per week, up to a maximum of four MED, where it was maintained. Tissue samples from the skin of the SKH-1 hairless mice were collected 72 h after the last UV irradiation. Regarding the actual estimated dose of UV penetration, it is discussed in Supplementary Note 1. All experimental protocols were approved by the Institutional Animal Care and Use Committee of the Biomedical Research Institute at Seoul National University Hospital. Following overnight fixation with 4% paraformaldehyde, mouse skin tissues were embedded in paraffin and sectioned using a microtome to produce 4-micron-thick sections. Paraffin was dissolved by baking the sections in a 65 °C oven for 30 min. The sections were then washed with Xylene I, II, 100, 95, 80, and 70% EtOH and distilled water for 5 min each to dissolve the paraffin and rehydrate the tissue sections. Antigen retrieval was performed by boiling the tissue sections in 100 mM sodium citrate for 5 min using a microwave oven. Following a 30-min cooling period, the samples were washed three times with PBS and permeabilized in PBS containing 0.5% Triton X-100 for 5 min. The sections were then washed three times with PBS and incubated in normal horse serum (1:30 dilution) for 30 min at room temperature. TOM20 primary antibody (1:50 dilution) was added to the sections and allowed to incubate overnight at 4 °C, followed by an additional hour at room temperature. The tissue sections were washed three times with PBS and incubated with secondary antibody (Anti-rabbit-IgG-Alexa488 [Invitrogen]) (1:200 dilution) for 1 h. After washing the section with PBS, the slides were mounted with Prolong Gold reagent.

### Western blot analysis

Whole-cell protein extracts were prepared from cells using RIPA lysis buffer (Merck Millipore, Billerica, MA, USA) mixed with a protease inhibitor cocktail (cOmplete mini™, Roche Applied Science, Rockford, IL, USA) and a phosphatase inhibitor cocktail (Sigma-Aldrich, St. Louis, MO, USA). The cell lysates were centrifuged at 12,000 g for 25 min at 4 °C, and the supernatants were collected. We quantified the total cell extract protein concentration using bicinchoninic acid assay reagent (Sigma-Aldrich, St. Louis, MO) and boiled the samples in SDS/PAGE sample buffer. Finally, we analyzed the samples via western blotting using antibodies against TOM20, TFAM, and alpha-tubulin.

### STED nanoscopy

For TOM20 cluster analysis on cells, we used a custom-built STED nanoscopy for both confocal and STED images. The fluorophore STAR635P was excited with light at 635 nm, which was selected by an excitation filter (z635/10x, Chroma) from a super-continuum light source generated through the 780-nm output from a Ti–Sapphire laser (80 MHz, < 100 fs, Mai Tai HP, Spectra-Physics) using a photonic crystal fiber (FemtoWhite, NKT photonics). Stimulated emission depletion was performed using the same laser, operating at 780 nm with a repetition rate of 80 MHz. We synchronized the two beams and adjusted the time delay between the excitation and STED beams to 180 ps. The STED beam had a doughnut shape at the focal plane after passing through a polymeric phase mask (VPP-1a, RPC Photonics). We used a 100X oil immersion type objective (HCX PL APO, 1.4 NA, Leica) to focus the two beams and collect the fluorescence signal. A piezo stage (NanoMax-TS, Thorlabs) was used to scan the sample area, and an avalanche photodiode (SPCM-AQR-14-FC, Perkin Elmer) was used to detect the fluorescence. To avoid saturation caused by the high power of the STED beam and eliminate the background signal, we arranged an IR block filter (FF01-720/SP-25, Semrock) and an emission filter (ET655LP, Chroma) in front of the detector. We controlled the image acquisition process with the Imspector software and obtained confocal and STED images at the same position.

For the quantitative analysis of TOM20 on tissues and TFAM on cells, we acquired both confocal and STED images on a Leica TCS SP8 microscope. We used a 488-nm excitation source for the Alexa488 fluorophore and a high-power Continuous wave (CW) laser at 592 nm to switch off the fluorescence and improve the spatial resolution. We controlled the image acquisition process with the Leica application suite X software. All STED setups were operated in 2D mode.

### Image analysis

To improve the S/N ratio of STED images and the accuracy of quantitative analysis, raw STED images were deconvoluted using *Imspector* and *Huygens*. The STED images were then quantitatively analyzed using the ImageJ software. The images were converted from RGB color to 8-bit format and adjusted using factors such as a band pass filter called Fast Fourier Transform algorithm and threshold. Information such as average TOM20 cluster size, TOM20 cluster density, and average number of TFAM per cell were statistically analyzed from the STED images. Specifically, we employed the ‘analysis particle’ plug-in to define the average size of TOM20 clusters across the entire area of highly resolved images. This plug-in was also utilized to count the average number of TFAMs per single cell. The area unit representing a single cell was determined by outlining the cell observed in bright-field imaging, and all TFAMs within a cell were counted. More than 30 cells were included in each group for generating quantitative analysis plots. Data from both confocal and STED measurements were used for comparison. For the quantitative analysis of TOM20 cluster density, expressed as a percentage unit, the area of highly resolved clusters in STED images was divided by the total area of the mitochondrial outer membrane determined from the confocal images. All images were obtained from different fields of view within the same sample.

### Immunogold stained electron microscopy sample preparation

Sub-cultured fibroblasts were plated on 100 π dishes and exposed to UV illumination. Harvested cells at the indicated time point were fixed with 4% paraformaldehyde in a pH 7.4 PBS buffer for 1 h. The pellet was infiltrated with a high concentration of sucrose and frozen in liquid nitrogen. Cryo-ultramicrotome sectioning was performed at -100 °C. Before immunogold staining, the sections were tested by immunofluorescence staining to confirm the proper functioning of the antigens on the section. Immunogold labeling was performed by incubating TOM20 primary antibodies and 1 nm gold conjugated secondary antibodies. Silver enhancement was performed using the HQ silver enhancement kit (Nanoprobs, Stony Brook, NY, USA) for 3 min to enlarge the gold nanoparticles to more than 10 nm using the seed growth approach. Images were captured using TEM (JEM series, JEOL, Tokyo, Japan).

### Ethics approval and consent to participate

Human tissues for primary cell culture were obtained under written informed consent and in accordance with an approved protocol by the Institutional Review Board of Seoul National University Hospital (IRB no. 1101–116-353). All experimental protocols for tissue were approved by the Institutional Animal Care and Use Committee of the Biomedical Research Institute at Seoul National University Hospital (IACUC No. 16–0075-S1A0). All experiments were performed in accordance with relevant guidelines and regulations. Especially, animal studies had performed in accordance with ARRIVE guidelines (https://arriveguidelines.org).

### Supplementary Information

Supplementary Figures.

## Data Availability

All relevant data are available within the article and its supplementary information files or from the corresponding authors upon reasonable request.
