# Supplementary material for: Uncovering the impact of UV radiation on mitochondria in dermal cells: a STED nanoscopy study
Source: Sci Rep. 2024 Apr 15;14:8675. doi: 10.1038/s41598-024-55778-z (PMC11018800; doi:10.1038/s41598-024-55778-z)
Supplement: Supplementary file 1 — Supplementary Figures. [file 41598_2024_55778_MOESM1_ESM.pdf]

Uncovering the Impact of UV Radiation on Mitochondria in Dermal Cells: A STED Nanoscopy Study

Hyung Jun Kim*, Seon-Pil Jin*, Jooyoun Kang, So Hyeon Bae, Jung Bae Son, Jang-Hee Oh, Hyewon Youn, Seong Keun Kim, Keon Wook Kang, and Jin Ho Chung

Supplementary Figures (total of six)

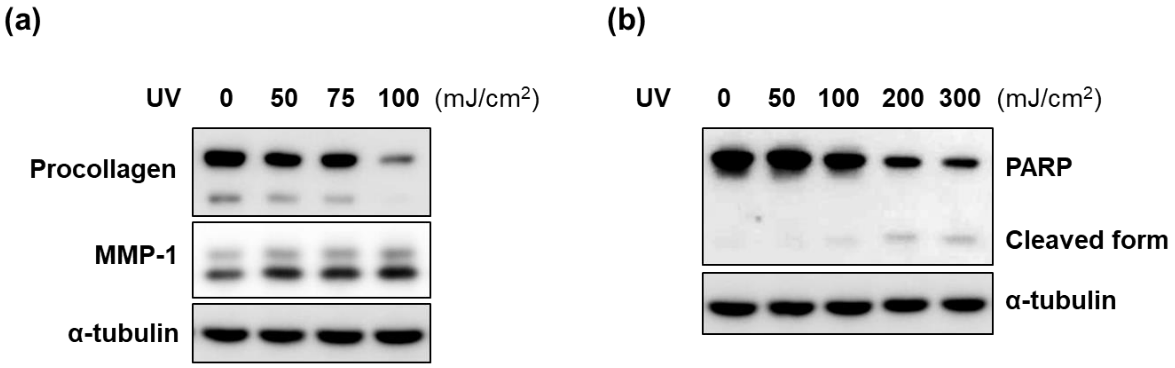

Supplementary Figure S1. 100 mJ/cm^2 UV irradiance shows marked biological effect without apoptosis. Primary human dermal fibroblasts starved with 0.25% fetal bovine serum for 24 h. Cells were irradiated with UV at the indicated dose. After 48 h, cell media (for procollagen and MMP-1) and lysate (for PARP) were harvested. (a) Decreased procollagen and increased MMP-1 were used as functional markers for the biological effect of UV. (b) Apoptosis was detected using cleaved form of PARP. Tubulin was a loading control.

Figure S1, original membrane scanned by a CCD camera

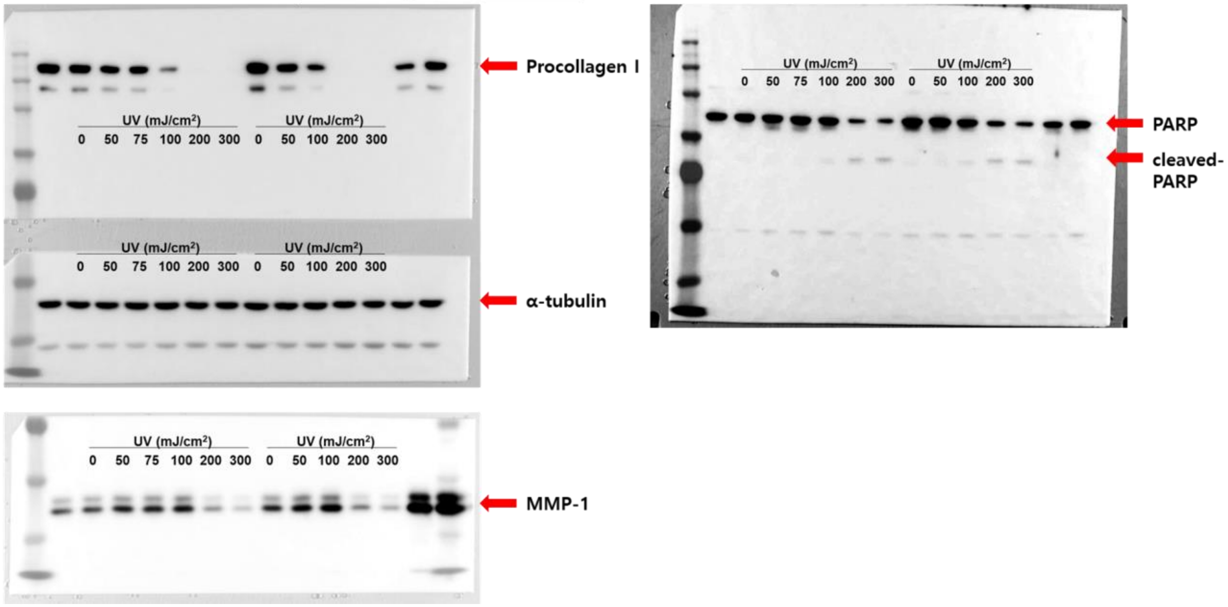

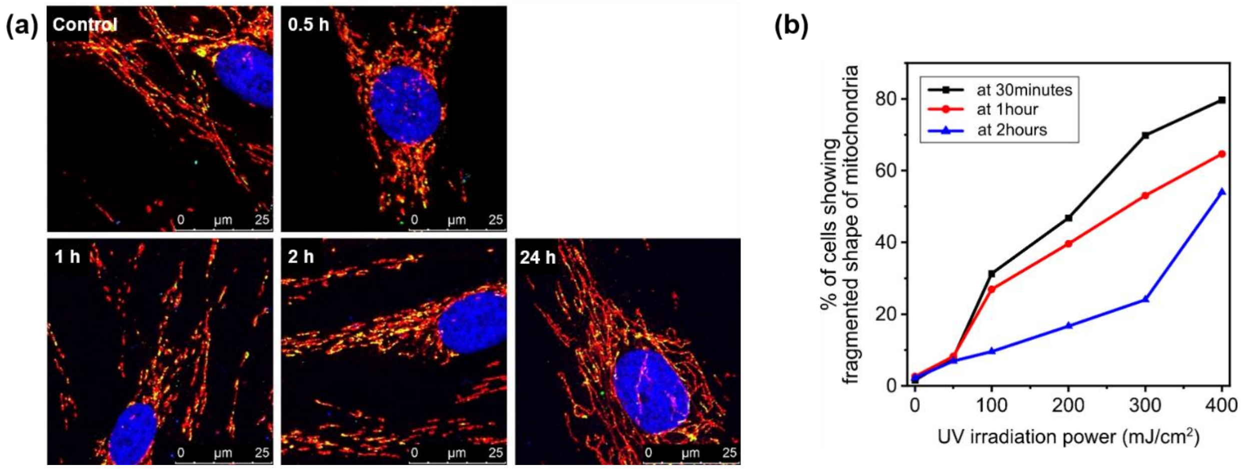

Supplementary Figure S2. Mitochondrial morphology changed from filamentous to spherical/fragmented shape after UV irradiation. (a) The mitochondrial structure was investigated through conventional confocal images in primary human dermal fibroblasts depending on the elapsed time after 100 mJ/cm^2 UV irradiation. TOM20 (red), TFAM (yellow), and nucleus (blue) were labeled as targets. (b) Percentage of cells showing the spherical/fragmented shape of mitochondria upon UV irradiance and the elapsed time. Over 160 cells were included in each group for generating quantitative analysis plots.

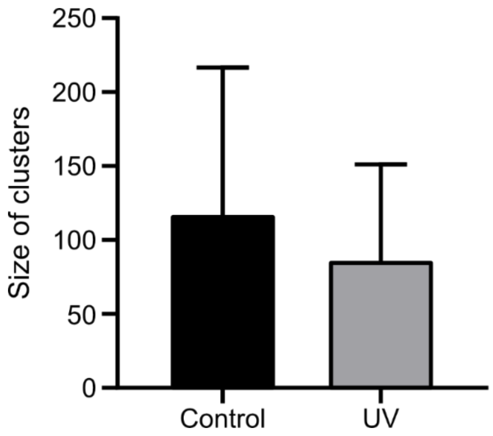

Supplementary Figure S3. The average diameter of TOM20 clusters is similar regardless of the experimental condition. All TOM20 clusters in a single 'field of view' were included in each group for generating quantitative analysis plots. Data are depicted as mean + SD.

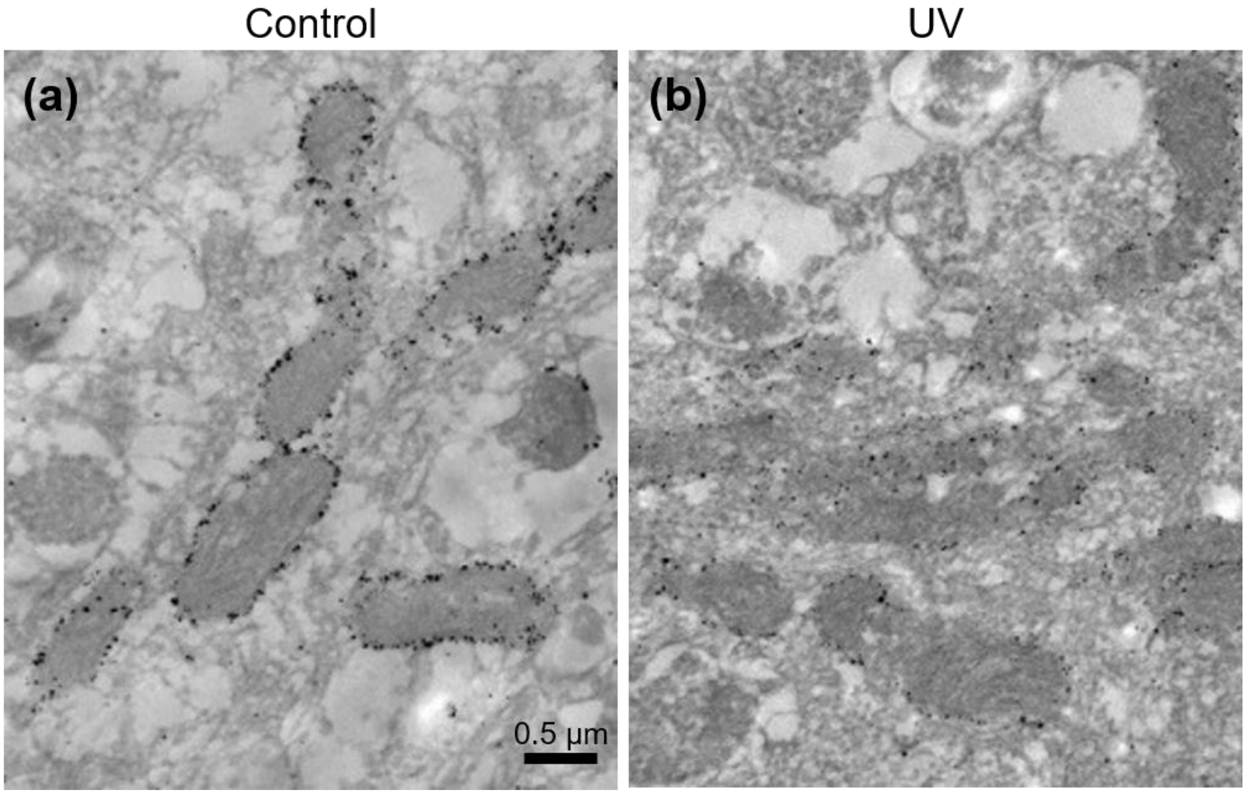

Supplementary Figure S4. Transmission electron microscopy (TEM) with immunogold labeling technique reveals decreased localization of TOM20 after UV irradiation. (a) Control sample. (b) UV-exposed sample. At each condition, cells were harvested and the cell pellet fixed with 4% paraformaldehyde. The section was made using a cryo-ultramicrotome, and the anti-TOM20 antibody was validated by immunofluorescence staining on this section. Then, immunogold labeling was performed with the silver enhancement technique.

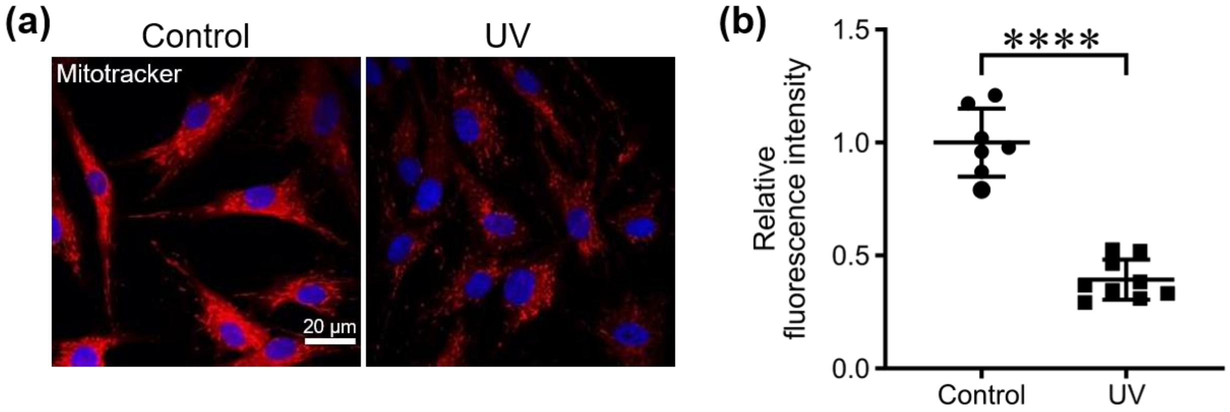

Supplementary Figure S5. UV irradiation decreases mitochondrial membrane potential, which can be measured by Mitotracker. (a) At each condition, cells were incubated with 200 nM of Mitotracker Deep Red® in serum-starved media for 30 min and then fixed with 4% formaldehyde. DAPI was used for nucleus staining. (b) The intensity analysis of Mitotracker dyes on mitochondria was performed using ImageJ (n=7-9 samples). ****$p < 0.0001$.

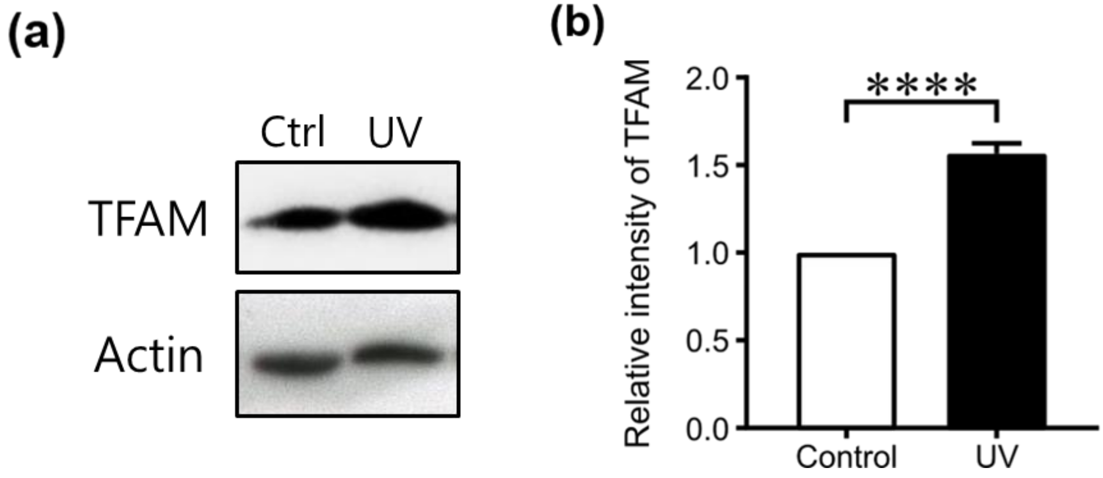

Supplementary Figure S6. The quantitative difference of TFAMs in cells was confirmed by Western blot. Primary human dermal fibroblasts starved with 0.25% fetal bovine serum for 24 h were used. At each condition, cells were harvested, and Western blot was performed with anti-TFAM antibody. (a) Increased TFAMs were used as functional markers for the biological effect of UV. Tubulin was a loading control. (b) The intensity analysis of TFAM from the Western blots was performed using ImageJ. **** p< 0.0001.

Figure S6, original film scan

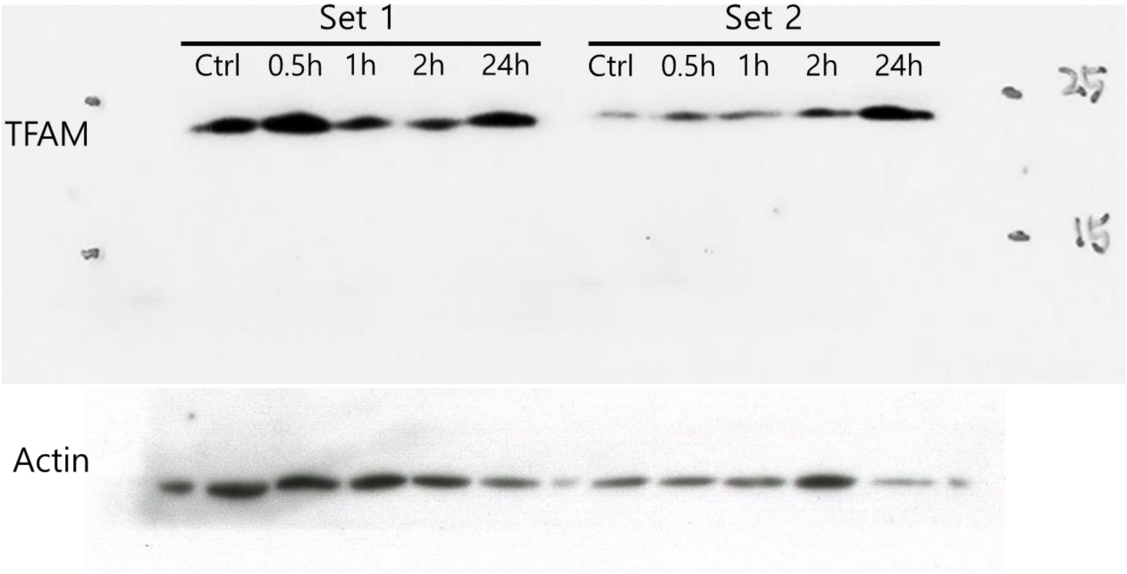

Conventional film-scanned images in Figure S6 cut the film and then scanned—no visible edges of the membrane but we can see complete blots in width. There is no other blot but TFAM in size smaller than 25 kDa.

Supplementary Note 1.

Theoretical estimation of effective intensity of UV illumination on tissues

The effective intensity of incident light to mouse skin tissues in our study may have indeed been influenced by various factors, including absorption and scattering coefficients for each skin layer, incident light angle, and lateral spread within skin layers. Specifically, UVB and UVA, spanning the wavelength range from 290 nm to 380 nm, comprise approximately 80% of the incident power, with around 37% effectively reaching the epidermis. Incident light below 10% effectively acts in the dermis, and the panniculus carnosus may be less responsive [1]. Despite these considerations, our chronic exposure to UV illumination at a power density of 100 mJ/cm^2 demonstrated aging-related changes in mitochondria, as observed in mouse tissues. This aligns with findings from a previous report [2] supporting the impact of UV exposure on biological responses, which reinforces the validity of our experimental outcomes.

References

1. Finlayson, L. et al. Depth Penetration of Light into Skin as a Function of Wavelength from 200 to 1000 nm. Photochem. Photobiol. 98, 974–981 (2022).

2. Salminen, A., Kaarniranta, K. & Kauppinen, A. Photoaging: UV radiation-induced inflammation and immunosuppression accelerate the aging process in the skin. Inflamm. Res. 71, 817–831 (2022).
